# Aqueous extract of Artemisia capillaris improves non-alcoholic fatty liver and obesity in mice induced by high-fat diet

**DOI:** 10.3389/fphar.2022.1084435

**Published:** 2022-11-28

**Authors:** Meng Liang, Mohan Huo, Yi Guo, Yuyi Zhang, Xiao Xiao, Jianwen Xv, Lixue Fang, Tianqi Li, Huan Wang, Siyu Dong, Xiaowen Jiang, Wenhui Yu

**Affiliations:** ^1^ Department of Veterinary Medicine, Northeast Agricultural University, Harbin, China; ^2^ Department of Life Sciences, Northeast Agricultural University, Harbin, China; ^3^ Institute of Chinese Veterinary Medicine, Northeast Agricultural University, Harbin, China; ^4^ Heilongjiang Provincial Key Laboratory for Prevention and Control of Common Animal Diseases, Northeast Agricultural University, Harbin, China

**Keywords:** aqueous extracts of Artemisia capillaris, NAFLD, lipid metabolism, PI3K/AKT, AMPK, SREBP-1c

## Abstract

Non-alcoholic fatty liver disease (NAFLD) is one of the most common chronic liver diseases and is a nutritional metabolic disease. *Artemisia capillaris* (AC) is the above-ground dried part of *Artemisia capillaris Thunb.* or *Artemisia scoparia Waldst. et Kit.*, a natural medicinal plant with pharmacological effects of heat-clearing and biliary-promoting. In order to evaluate the therapeutic effect of Artemisia capillaris on NAFLD and obesity, experiments were conducted using aqueous extracts of Artemisia capillaris (WAC) to intervene in NAFLD models *in vivo* and *in vitro*. *In vivo* experiments were performed using HFD-fed (high fat diet) C57BL/6 mice to induce NAFLD model, and *in vitro* experiments were performed using oleic acid to induce HepG2 cells to construct NAFLD cell model. H.E. staining and oil red O staining of liver tissue were used to observe hepatocytes. Blood biochemistry analyzer was used to detect serum lipid levels in mice. The drug targets and mechanism of action of AC to improve NAFLD were investigated by western blotting, qRT-PCR and immunofluorescence. The results showed that C57BL/6 mice fed HFD continuously for 16 weeks met the criteria for NAFLD in terms of lipid index and hepatocyte fat accumulation. WAC was able to reverse the elevation of serum lipid levels induced by high-fat diet in mice. WAC promoted the phosphorylation levels of PI3K/AKT and AMPK in liver and HepG2 cells of NAFLD mice, inhibited SREBP-1c expression, reduced TG and lipogenesis, and decreased lipid accumulation. In summary, WAC extract activates PI3K/AKT pathway, reduces SREBP-1c protein expression by promoting AMPK phosphorylation, and decreases fatty acid synthesis and TG content in hepatocytes. AC can be used as a potential health herb to improve NAFLD and obesity.

## 1 Introduction

Non-alcoholic fatty liver disease (NAFLD) is a clinicopathologic syndrome of hepatic steatosis. It is characterized by excessive accumulation of lipids, mainly triglycerides (TG), in hepatocytes. However, NAFLD does not include a history of excessive alcohol consumption and definite liver damage factors (Cohen et al., 2011; Chalasani et al., 2018). NAFLD is a chronic nutritional metabolic disease that can develop into hepatitis, cirrhosis, and even liver cancer (Diehl and Day, 2017; Huang et al., 2021; Ratziu et al., 2022). Studies have shown that the incidence of NAFLD has shown a high development worldwide (Lonardo et al., 2016). It is closely associated with other metabolic diseases such as obesity, type 2 diabetes, dyslipidemia and hypertension, so NAFLD has become a global health problem of concern (Chalasani et al., 2018). Currently, clinical treatment is mostly carried out with lipid-lowering drugs, antioxidants and insulin sensitizers, but long-term use of these drugs produces toxic side effects. Natural medicines as alternative therapies have their unique advantages in the treatment of NAFLD, and there are some herbal medicines that have potential applications in the treatment of NAFLD.

The occurrence of NAFLD is closely related to insulin resistance (IR). When IR occurs in the body, in order to meet the body’s energy supply needs, fat becomes the main mode of energy supply. After the increased breakdown of peripheral adipose tissue in the body, the uptake of free fatty acids by hepatocytes increases and they are deposited in the form of TG in the cells, and eventually NAFLD occurs. The phosphatidylinositol-3-kinase (PI3K) signaling pathway (PI3K/AKT) is the classical hepatic insulin signaling pathway (Fukuda et al., 2016). Activation of the PI3K/AKT transduction pathway improves IR and alleviates NAFLD. AMP-activated protein kinase (AMPK) is an important factor in the development of NAFLD and regulation of hepatic lipid metabolism. Activation of AMPK has an important regulatory role on lipid metabolism (Kamikubo et al., 2016; Garcia et al., 2019). Activated AMPK promotes lipid catabolism and ATP production, reduces mitochondrial ATP consumption to maintain the steady state of cellular energy, and regulates the activity of lipid metabolism-related enzymes to regulate fatty acid synthesis and oxidation in the liver (Hardie, 2015; Garcia and Shaw, 2017). AMPK inhibits sterol-regulatory element binding proteins-1c (SREBP-1c) activity, inhibits lipid synthesis, and reduces lipid deposition in the liver (Day et al., 2017). SREBP is an endoplasmic reticulum (ER) membrane-bound transcription factor that activates genes encoding fatty acid synthesis and regulating cholesterol (Horton et al., 2002). SREBP-1c, one of the isoforms of the SREBP family, is a key transcription factor with the function of enhancing the expression of genes related to hepatic adipogenesis (Irimia et al., 2017). It is mainly distributed in the liver and adipose tissue. Overexpression of SREBP-1c will cause lipid metabolism disorders. Activation of AMPK can downregulate SREBP-1c expression, control fatty acid oxidation (Samovski et al., 2015). TG and TC production in the liver, reduce lipid accumulation in hepatocytes, improve lipid metabolism disorders in the liver, and alleviate NAFLD (Tang et al., 2016).

Artemisia capillaris (AC) is a dried above-ground part of Artemisia capillaris Thunb. or Artemisia scoparia Waldst. et Kit. which is commonly used in traditional medicine for the treatment of clearing heat and gallbladder. It mainly contains coumarins, flavonoids, organic acids, volatile oils, terpenoids and other chemical components (Yuan et al., 2017). Recent studies have found that, in addition to its pharmacological effects such as choleretic and hepatoprotective, AC also has a variety of pharmacological activities such as antipyretic, anti-inflammatory, analgesic, and regulating lipid metabolism (Hsueh et al., 2021)。Organic acids such as chlorogenic acid and caffeic acid contained in AC exhibited potential anti-obesity effects in mice induced by high-fat diet (HFD) (Cho et al., 2010). In addition, Ethanol extract of Artemisia scoparia (Galluzzi et al., 2018) has a modulating effect on lipid metabolism. The addition of SCO to HFD significantly reduced the levels of circulating glycerol and free fatty acids, and *in vitro* experiments revealed that SCO attenuated the lipolytic effect in TNF-α-induced cultured adipocytes (Boudreau et al., 2018).

In the present study, we evaluated the effects of WAC on a high-fat diet-induced mouse model of NAFLD and an oleic acid-induced HepG2 cell model. Then we explored the mechanisms by which WAC improves lipid accumulation *in vivo* and *in vitro*.

## 2 Materials and methods

### 2.1 Preparation of WAC

The air-dried AC used in this study, produced in Gansu Province, China, was purchased from Harbin Songshantang Pharmaceutical Company, China. AC was identified by a professor from the Institute of Chinese Veterinary Medicine, Northeastern Agricultural University. Weigh 100 g of dried AC, add 800 ml of distilled water, soak for 30 min at room temperature, decoct for 1 h, and collect the decoction solution. Then, 600 ml of distilled water was added and the decoction was decocted again for 1 h. The decoction was collected. The decoction solution collected twice was mixed, and the decoction solution was filtered, centrifuged to remove insoluble substances, and lyophilized at -50°C for 48 h to a powdered solid (Huang et al., 2022). The average yield of AC was 15.6% (W/W). The lyophilized product was stored at -20°C. For cellular experiments, WAC was dissolved in DMEM and filtered (0.22 μm).

### 2.2 Identification of WAC active ingredients by LC-MS

WAC (100 mg) were individually grounded with liquid nitrogen and the homogenate was resuspended with prechilled 80% methanol by well vortex. The samples were incubated on ice for 5 min and then were centrifuged at 15,000 g, 4°C for 20 min. Some of supernatant was diluted to final concentration containing 53% methanol by LC-MS grade water. The samples were subsequently transferred to a fresh Eppendorf tube and then were centrifuged at 15000 g, 4°C for 20 min (Want et al., 2013). Finally, the supernatant was injected into the LC-MS/MS system analysis LC-MS/MS analyses were performed using an ExionLCTM AD system (SCIEX) coupled with a QTRAP® 6,500 + mass spectrometer (SCIEX). Samples were injected onto a Xselect HSS T3 (2.1 × 150 mm, 2.5 μm) using a 20-min linear gradient at a flow rate of 0.4 ml/min for the positive/negative polarity mode. The eluents were eluent A (0.1% Formic acid-water) and eluent B (0.1%Formic acid-acetonitrile) (Luo et al., 2015). The solvent gradient was set as follows: 2% B, 2 min; 2–100% B, 15.0 min; 100% B, 17.0 min; 100–2% B, 17.1 min;2% B, 20 min. QTRAP® 6,500 + mass spectrometer was operated in positive polarity mode with Curtain Gas of 35 psi, Collision Gas of Medium, Ion Spray Voltage of 5500V, Temperature of 550°C, Ion Source Gas of 1:60, Ion Source Gas of 2:60. QTRAP® 6,500 + mass spectrometer was operated in negative polarity mode with Curtain Gas of 35 psi, Collision Gas of Medium, Ion Spray Voltage of -4500V, Temperature of 550°C, Ion Source Gas of 1:60, Ion Source Gas of 2:60.

### 2.3 Experimental grouping and sample collection

Five-week-old male C57BL/6 mice were purchased from Changsheng Biotechnology (Changsheng, Liaoning, China). Animal experimental design strictly followed the guidelines of the International Association for the Evaluation and Accreditation of Laboratory Animal Care and was licensed by the Research Animal Protection Committee of Northeast Agricultural University. Mice were housed at 25 ± 1°C and in humidity-controlled chambers with natural light and dark cycles. After a 1-week adaptation period, mice were randomly divided into two groups: normal diet (ND) group (*n* = 6); and high-fat diet (HFD) group (*n* = 12). The nutritional analysis values and energy supply ratios of the high-fat diet are shown in Tables 1, 2, respectively. Mice in the high-fat diet group at week 16 formed a model of NAFLD (Li et al., 2022). Twelve mice in the high-fat diet group were randomly divided into two groups: the NAFLD model group (*n* = 6), and the NAFLD + WAC group (HFD + WAC, 50 mg/kg/d, WAC gavage twice a week, *n* = 6). WAC gavage lasted for 6 weeks. The experimental period was 22 weeks. The doses administered in this experiment were selected based on the experiments of Choi (Choi et al., 2013). All mice were fed and watered *ad libitum*, and body weight and food intake were recorded weekly. At the end of the animal experimental period, all mice were fasted and watered *ad libitum* for 12 h. Then, blood was collected from the eyes after anesthesia with ether, and serum was obtained by centrifugation at 3,000 rpm for 5 min at 4°C. Livers were immediately excised, collected and stored at -80°C for analysis, or stored in 4% paraformaldehyde for histological studies.

**TABLE 1:** Nutritional analysis values.

Feed components	Ratio (%)
Crude protein	19.2
Crude fat	14.4
Crude fiber	3.8
Crude ash content	4.0
Water content	9.2
Calcium	0.82
Total phosphorus	0.69

**TABLE 2:** Energy supply ratio.

Energy value	Ratio (%)
Protein	20.8
Fat	34.9
Carbaohydrate	44.3

### 2.4 Detection of major lipid indicators and glucose in blood and liver tissues

Serum TC, TG, Glucose, HDL and LDL levels were measured by a fully automated biochemical analyzer, and liver TC and TG levels were measured by a commercially available kit (Jiancheng, Nanjing, China). The liver tissue samples were accurately weighed, mechanically homogenized under ice and water bath conditions, centrifuged at 2,500 rpm for 10 min, and the supernatant was extracted and used to assay TG and TC levels.

### 2.5 Histological tests for liver pathology

Immediately after execution of the mice, liver tissue was fixed in 4% paraformaldehyde, paraffin-embedded, and sections were stained for H&E. The tissues were sectioned using a fcryostat and stained for Oil Red O. Light was observed using an optical microscope (Nikon, Tokyo, Japan).

### 2.6 Western blot analysis

Total protein samples were prepared using RIPA lysis buffer containing protease inhibitors and phosphatase inhibitors and quantified by the BCA protein assay kit. Equal amounts of proteins were separated by sodium dodecyl sulfate-polyacrylamide gel electrophoresis (SDS-PAGE). The molecular weight region of the target protein indicated by the pre-stained Marker was cut off and transferred to PVDF membrane or NC membrane, and the skimmed milk powder was closed at room temperature for 2 hours, and the primary antibody was added at the end of closure and incubated in the refrigerator at 4°C overnight. The primary antibodies are shown in Table 4. Then incubation with secondary antibody IgG (1:10,000) and preparation of ECL luminescent solution (meilunbio, Dalian, China) was performed for strip exposure. The strips were exposed using a 5,200 Tanon imaging system (Tanon science and technology, Shanghai, China). The developed protein bands were further quantified by ImageJ software and then compared with the grayscale values of the internal reference gene β-tubulin to calculate the relative expression levels of the target proteins measured in each of the above experimental groups.

**TABLE 4:** Antibodies and reagents.

Antibodies and Reagents	Producer
Rabbit anti-PI3K(A11402, 1:500)	Abclonal, Wuhan, China
Rabbit anti-p-PI3K(A4992, 1:500)	Abclonal, Wuhan, China
Rabbit anti-AKT(A18120, 1:500)	Abclonal, Wuhan, China
Rabbit anti-p-AKT(AP1068, 1:500)	Abclonal, Wuhan, China
Rabbit anti-AMPK(WL02254, 1:1000)	Wanleibio, Shenyang, China
Rabbit anti-p-AMPK(WL05103, 1:1000)	Wanleibio, Shenyang, China
Rabbit anti-SREBP-1c(WL02093, 1:1000)	Biodragon, Suzhou, China
Rabbit anti-SCD1(A15606, 1:1000)	Abclonal, Wuhan, China
Rabbit anti-FAS(WL03376, 1:1000)	Wanleibio, Shenyang, China
Rabbit anti-DGAT2(A13891, 1:1000)	Abclonal, Wuhan, China
Rabbit anti-β-tubulin(AC008, 1:10000)	Abclonal, Wuhan, China
Rabbit anti-SREBP-1c(A15586, 1:200)	Abclonal, Wuhan, China
Goat Anti-rabbit IgG/FITC antibody(bs-0295G-FITC)	Bioss, Beijing, China
BODIPY 493/503	yuanye, Shanghai, China
DAPI staining solution(BL105A)	Biosharp, Hefei, China
Fatostatin (125B11)	MCE, Shanghai, China
BioRT Master HiSensi cDNA First Strand Synthesis kit	Boster, USA
BioEasy Master Mix (SYBR Green)(BSB25L1B)	Boster, USA
High glucose DMEM(cat.no.SH30022.01)	HyClone, Shanghai, China
Fetal Bovine Serum(WHRTNA101-1)	Wohong, Shijiazhuang, China
Penicillin-Streptomycin mixture	Solarbio, Beijing, China
Oleic acid	yuanye, Shanghai, China
High fat diet and normal diet	Xiaoshuyoutai, Beijing, China

### 2.7 HepG2 cell culture and drug treatment

HepG2 cells were provided by Dr. Zhang, College of Food, Northeastern Agricultural University. HepG2 cells were cultured in DMEM medium supplemented with 10% fetal bovine serum and 1% penicillin/streptomycin at 37°C and 5% CO_2_ atmosphere. In the experiments, cells were starved in DMEM without FBS for 12 h. Subsequently, cells were cultured with DMEM containing FBS (negative control), OA-BSA complex (0.5 mM) (Xie et al., 2016) alone for 24 h to develop steatosis, and OA (0.5 mM) and WAC (1.5 mg/ml) were co-treated for 24 h. Next, cells were collected for subsequent experimental analysis. OA-BSA complexes were prepared by first dissolving OA in NaOH at 75°C by saponification and then adding the saponified OA to the medium containing 5% BSA and heating at 55°C until the solution became clear and transparent, resulting in a final concentration of 0.5 mM OA-BSA complexes.

### 2.8 HepG2 cell viability assay

Cell viability was assessed by cck-8 assay. HepG2 cells were inoculated at a density of 5 × 104 per well in 96-well plates with 100 μL of medium per well. Six replicate wells were set up and HepG2 cells were pre-cultured in an incubator at 37°C for 24 h. Then OA was co-treated with WAC (0.5 mg/ml, 1 mg/ml, 1.5 mg/ml, 2 mg/ml, 2.5 mg/ml, 3 mg/ml) for 24 h. 10 μL of cck-8 working solution was added to each well, protected from light, and incubated for 0.5 h in an incubator at 37°C. The absorbance at 450 nm was measured by an enzyme marker, and the cell survival rate (%) was calculated relative to the control group.

### 2.9 HepG2 cell lipid droplet observation

Oil Red O staining was used to assess the level of intracellular lipid droplets. The cells were washed 3 times with PBS, fixed with 4% paraformaldehyde for 10 min, and washed 3 times with PBS. The appropriate amount of staining washing solution was added to cover cells for 20 s and then aspirated and stained with modified Oil Red O staining solution for 20 min, and then the modified Oil Red O staining solution was removed with staining washing solution and washed with PBS. Finally, stained HepG2 cells were observed with an E100 light microscope (Nikon, Tokyo, Japan). For BODIPY staining, HepG2 cells were first inoculated in 24-well plates and treated with OA and WAC when the density reached 60%–70% for 24 h. Cells were fixed with 4% paraformaldehyde PBS for 1 h at room temperature, then washed 3 times with PBS for 5 min each, stained with 4 μM BODIPY493/503 at 37°C for 30 min, washed with PBS for 3 times, re-stained with DAPI for 5 min, and washed with PBS for 3 times. After blocking with PBS, the cell plates were placed on an inverted fluorescence microscope and the fluorescence signal was acquired and quantified using Image-Pro Plus software (version 3.0, Media Cybernetics, Bethesda, MD, United States) and the same parameters.

### 2.10 Detection of TC and TG content in HepG2 cells

Commercially available kits (Nanjing Jiancheng Bioengineering Co., Ltd. Nanjing, China) were used to determine intracellular TC and TG levels and to assess the extent of lipid accumulation. TG and TC levels were measured according to the manufacturer’s instructions, and protein concentrations were determined by the Enhanced BCA Protein Assay Kit. Then, lipid levels/protein concentrations were calculated.

### 2.11 Immunofluorescence assay

HepG2 cells were plated in 24-well plates and treated with OA and WAC when the density reached 60%–70% for 24 h. Cells were fixed with 4% paraformaldehyde, washed, permeabilized, and closed. Cells were incubated with SREBP-1c (1:200) primary antibody overnight at 4°C in the refrigerator. Secondary antibody goat anti-rabbit IgG HampL/HRP (1:200) was added to the cells for 1 h. The nuclei were stained with DAPI (BL105A, Biosharp, Hefei, China) for 5 min. After blocking with PBS, the cell plates were placed on an inverted fluorescence microscope and the fluorescence signal was acquired and quantified using Image-Pro Plus software (version 3.0, Media Cybernetics, Bethesda, MD, United States) and the same parameters.

### 2.12 qRT-PCR analysis

0.1 g of liver tissue was weighed, total RNA was extracted and RNA concentration was detected according to the kit. RNA was reverse transcribed into cDNA using the kit, primers were synthesized by Sangon Biotech (Sangon Biotech Co., Ltd. Shanghai, China), primer design is shown in Table 3 qRT-PCR was performed using SYBR Green in a Light Cycler@480 System (Roche, Switzerland). reaction conditions were: pre-denaturation at 95°C for 10 min; denaturation at 95°C for 15 s, denaturation at 60°C for 1 min, 40 cycles; and finally melting reaction. The mRNA expression level was quantified using β-actin as the control. The calculation method used was 2^−ΔΔCt^ method.

**TABLE 3:** mRNA primer sequences.

Name	Forward 5–3’	Reverse 5–3’
FASN	TAA​AGC​ATG​ACC​TCG​TGA​TGA​A	GAA​GTT​CAG​TGA​GGC​GTA​GTA​G
ACC	GGC​CAG​TGC​TAT​GCT​GAG​AT	AGG​GTC​AAG​TGC​TGC​TCC​A
SREBP-1c	GCT​ACC​GGT​CTT​CTA​TCA​ATG​A	CGC​AAG​ACA​GCA​GAT​TTA​TTC​A
SCD-1	AAC​ATT​CAA​TCC​CGG​GAG​AAT​A	GAA​ACT​TTC​TTC​CGG​TCG​TAA​G

### 2.13 Statistical analysis of data

Data were expressed as mean ± standard deviation, and statistical analysis was performed using SPSS software 22.0. Statistical differences in quantitative data between groups and significance of nonparametric comparisons between groups were analyzed using one-way ANOVA and Tukey’s test. Plots were performed using Origin 2019. *p* < 0.05 was considered statistically significant.

## 3 Results

### 3.1 LC-MS analysis of WAC

**FIGURE 1:**
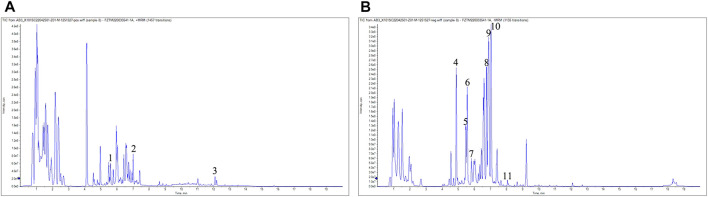
LC-MS analysis. **(A)** positive mode **(B)** negative mode. 1) 1-Caffeoylquinic acid (rt = 5.46 min), 2) Isoferulic Acid (rt = 7.008 min),3) Costunolide (rt = 12.083 min), 4) Neochlorogenic acid (rt = 4.879 min), 5) Chlorogenic Acid (rt = 5.37 min), 6) 3-O-Feruloylquinic acid (rt = 5.55 min), 7) Caffeic acid (rt = 5.801 min), 8) Isochlorogenic acid B (rt = 6.768 min), 9) 1,5-Dicaffeoylquinic acid (rt = 7.676 min), 10) Isochlorogenic acid C (rt = 7.053 min), 11) Scoparone (rt = 7.935 min).

As shown in Figure 1A,B, 11 compounds were characterized by LC/MS, including 1) 1-Caffeoylquinic acid (rt = 5.46 min), 2) Isoferulic Acid (rt = 7.008 min), 3) Costunolide (rt = 12.083 min), 4) Neochlorogenic acid (rt = 4.879 min), 5) Chlorogenic Acid (rt = 5.37 min), 6) 3-O-Feruloylquinic acid (rt = 5.55 min), 7) Caffeic acid (rt = 5.801 min), 8) Isochlorogenic acid B (rt = 6.768 min), 9) 1,5-Dicaffeoylquinic acid (rt = 7.676 min), 10) Isochlorogenic acid C (rt = 7.053 min), and 11) Scoparone (rt = 7.935 min). The WAC extract samples used in this study contained the active ingredients described in previous reports (Dai et al., 2021; Hsueh et al., 2021).

### 3.2 WAC alleviates high fat diet-induced obesity and hepatic steatosis in non-alcoholic fatty liver disease mice

**FIGURE 2:**
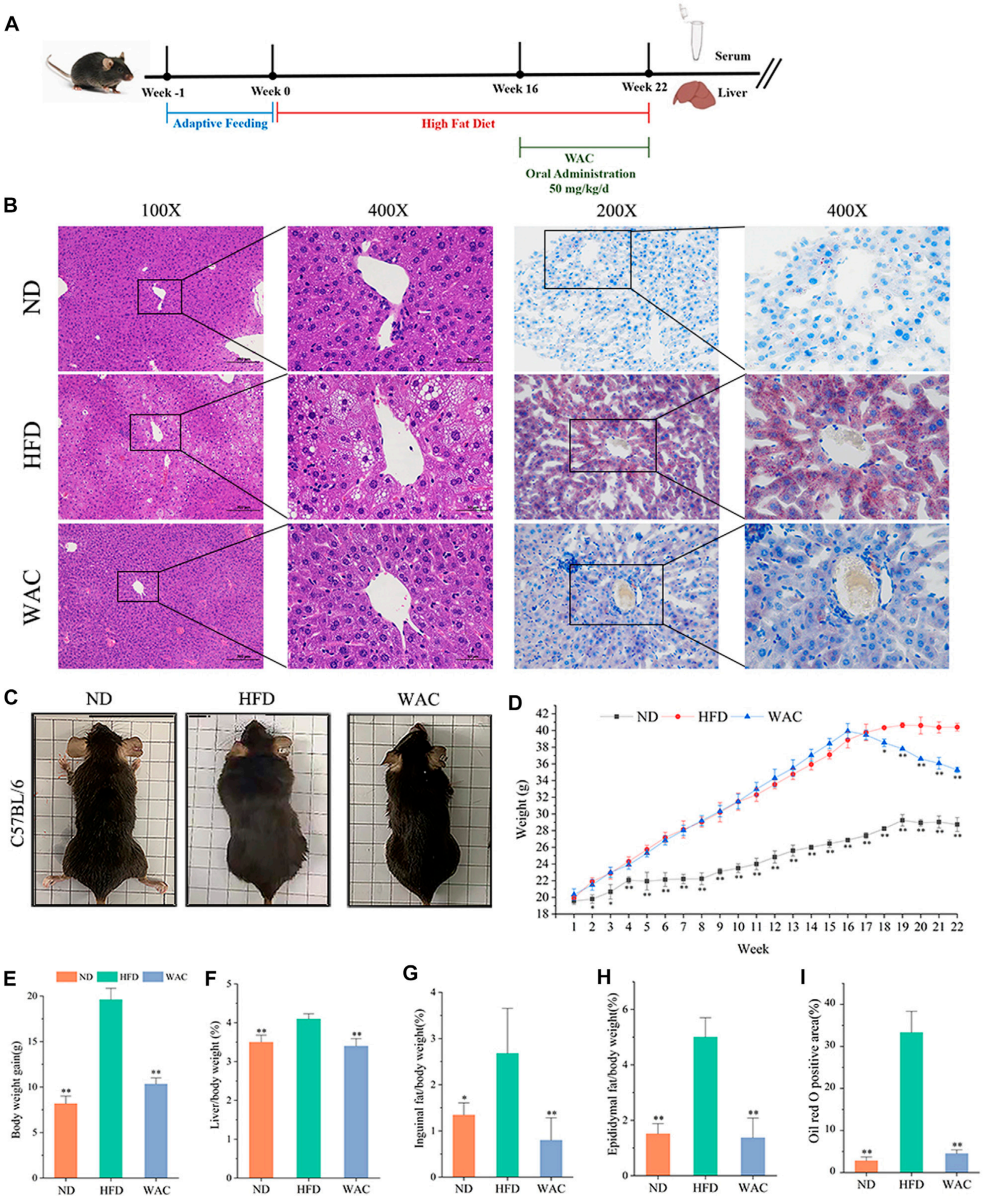
Effect of WAC on obesity and hepatic steatosis in HFD-induced NAFLD mice. **(A)** Animal experimentation process. **(B)** Pictures of liver morphology, HE staining and oil red O staining of liver sections after 6 weeks of WAC gavage. **(C)** Body condition of mice in each group after 6 weeks of WAC gavage. **(D)** Body weight changes in mice over 22 weeks. **(E)** Body weight gain in mice over 22 weeks. **(F)** Mouse liver index. **(G)** Index of inguinal adipose tissue in mice. **(H)** Adipose tissue index of mouse epididymis. **(I)** The positive areas of oil-red O staining were analyzed using ImageJ. Compared with HFD, * indicates *p* < 0.05 and ** indicates *p* < 0.01.

To determine the effect of WAC on NAFLD *in vivo*, a mouse model of NAFLD was constructed by feeding mice with HFD for 16 weeks, and WAC was given by gavage at 50 mg/kg/d for the next 6 weeks (Figure 2A). In terms of histopathology, WAC treatment significantly improved fat accumulation in the liver of HFD-fed mice (Figures 2B,I). Mice fed with HFD showed higher weight gain. weight gain was reduced after WAC treatment (Figures 2C–E). The liver index, inguinal adiposity index and epididymal adiposity index were significantly increased in HFD-fed mice, and the above indices were significantly reduced after 6 weeks of WAC treatment. HFD enhanced the adiposity index and WAC treatment reduced the adiposity index, suggesting that the weight loss was associated with a reduction in the amount of adiposity (Figures 2F–H). These results suggest that WAC treatment alleviates obesity and hepatic steatosis in HFD-induced NAFLD mice.

### 3.3 WAC ameliorates lipid metabolism disorders in high fat diet-induced non-alcoholic fatty liver disease mice

**FIGURE 3:**
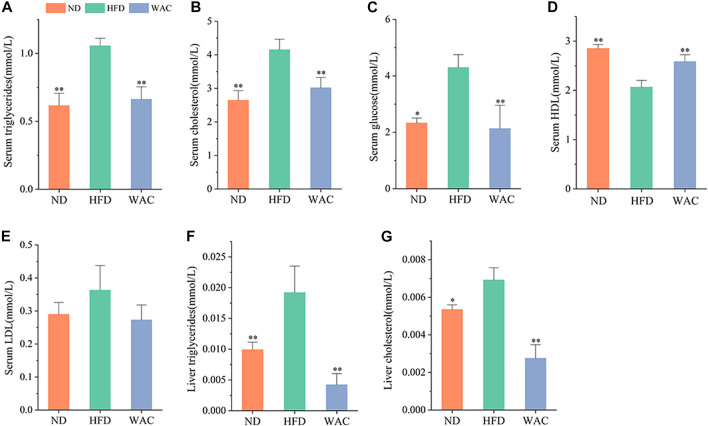
Effect of WAC on metabolic disorders in HFD-induced NAFLD mice. **(A)** Serum triglyceride levels. **(B)** Serum cholesterol levels. **(C)** Serum glucose levels. **(D)** Serum HDL levels. **(E)** Serum LDL levels. **(F)** Liver triglyceride levels in mice. **(G)** Liver cholesterol levels in mice. Compared with HFD, * indicates *p* < 0.05 and ** indicates *p* < 0.01.

Excessive accumulation of hepatic lipids is due to a dysregulation of lipid metabolic homeostasis, which ultimately leads to hepatic steatosis. Mice fed HFD exhibited significantly higher serum TG and TC levels compared to mice in the ND group, and WAC treatment restored serum triglyceride and cholesterol levels to normal levels (Figures 3A,B). Elevated serum glucose in mice fed HFD was also reversed by WAC, restoring normal levels (Figure 3C), suggesting that WAC may regulate glucose metabolism in mice. Compared with mice in the ND group, serum levels of HDL were reduced and LDL levels were increased in mice fed HFD, and both reduced HDL levels and increased LDL levels were reversed after 6 weeks of WAC treatment (Figures 3D,E). In addition, the liver of HFD-induced NAFLD mice exhibited elevated TG and TC levels, which were significantly reduced after WAC treatment (Figures 3F,G). These results suggest that WAC treatment ameliorates metabolic disorders in HFD-induced NAFLD mice.

### 3.4 WAC ameliorates OA-induced lipid accumulation in HepG2 cells

**FIGURE 4:**
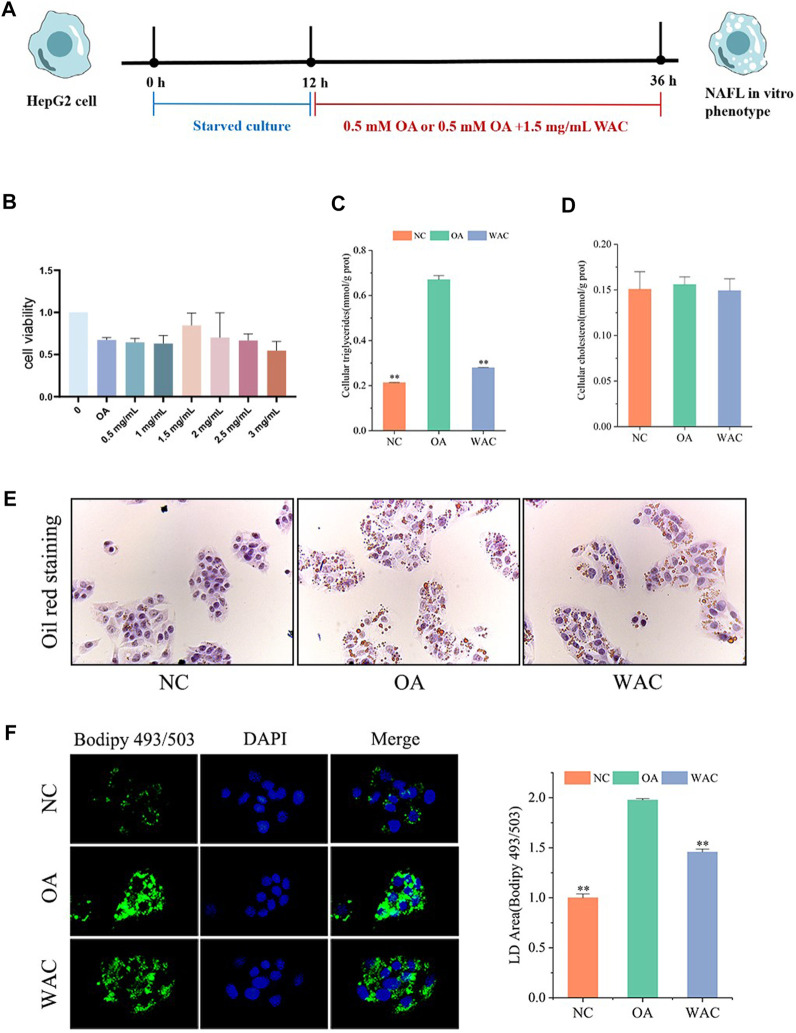
Effect of WAC on lipid accumulation in OA-induced HepG2 cells. **(A)** Cellular experimental procedures. **(B)** Cell viability. **(C,D)** Triglyceride and cholesterol levels in HepG2 cells. **(E)** Graph of oil red O staining results. **(F)** Graph of BODIPY staining results. Compared with OA, * indicates *p* < 0.05 and ** indicates *p* < 0.01.

The cytotoxic effect of WAC was determined by cck-8, and 1.5 mg/ml was selected as the WAC concentration for the following *in vitro* experiments (Figure 4B). *In vitro*, lipid accumulation in HepG2 cells was induced with 0.5 mM OA. The effect of WAC on lipid accumulation in HepG2 cells was assayed by co-treatment of HepG2 cells with WAC and OA (Figure 4A). Compared with the NC group, the TG content of HepG2 cells in the OA group was significantly higher, whereas the TG content was significantly lower after treatment of HepG2 cells with WAC (Figure 4C). In contrast to the *in vivo* results, the OA-induced TC of HepG2 cells did not change, nor did WAC treatment cause changes in the TC of HepG2 cells (Figure 4D). The results of cytosolic oil red O staining showed a significant increase in lipid droplets in HepG2 cells in the OA group compared to the NC group. There was a decrease in lipid droplets in HepG2 cells in the WAC group compared to the OA group (Figure 4E). After HepG2 cells were incubated with OA for 24 h, lipid accumulation was detected by staining with BODIPY493/503 (a specific lipophilic probe), which showed that OA significantly promoted lipid accumulation in HepG2 cells, and the green fluorescence intensity of HepG2 cells in the WAC group was diminished (Figure 4F). These results suggest that *in vitro*, WAC ameliorates OA-induced lipid accumulation in HepG2 cells.

### 3.5 WAC activates PI3K/AKT and AMPK *in vivo*

**FIGURE 5:**
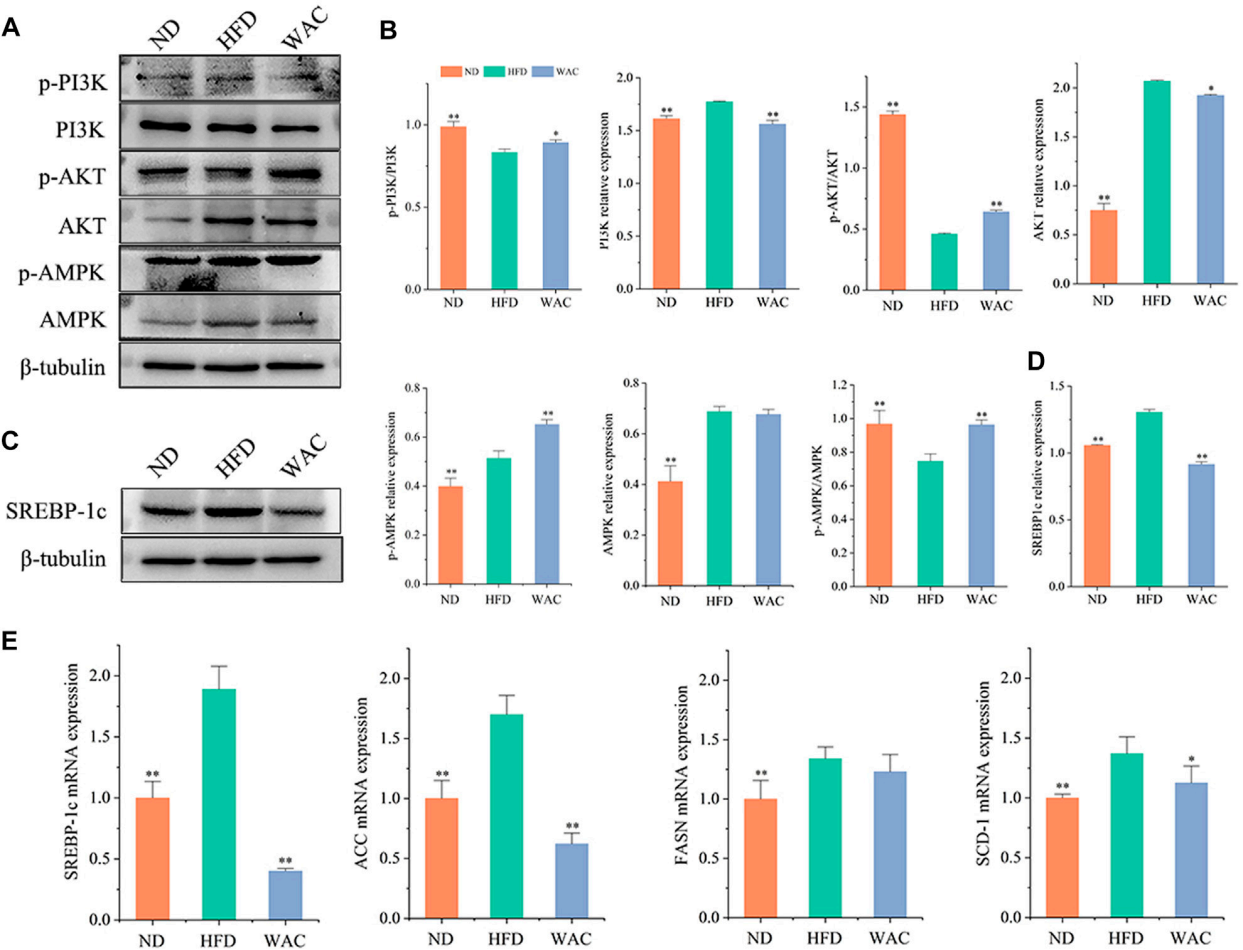
Effect of WAC on PI3K, AKT, AMPK phosphorylation and SREBP-1c protein expression in HFD-induced NAFLD mice. **(A,B)** Phosphorylation and total protein levels of PI3K, AKT, and AMPK in mouse liver. **(C,D)** Protein levels of SREBP-1c in mouse liver. **(E)** The mRNA levels of mouse liver FASN, ACC, SREBP-1c and SCD-1. Compared with HFD, * indicates *p* < 0.05 and ** indicates *p* < 0.01.

Total PI3K and AKT and their phosphorylation levels, AMPK and its phosphorylation levels were detected *in vivo* by western blotting. Mice in the HFD group had elevated PI3K and AKT protein levels, decreased phosphorylated PI3K and AKT protein levels, decreased p-PI3K/PI3K and p-AKT/AKT levels, and significantly increased AMPK and p-AMPK protein levels in the liver. Mice in the WAC group had decreased PI3K and AKT protein levels, increased phosphorylated PI3K and AKT protein levels, increased p-PI3K/PI3K and p-AKT/AKT levels, and increased p-AMPK/AMPK (Figures 5A,B). Liver SREBP-1c protein levels were significantly elevated in mice in the HFD group, and WAC treatment significantly decreased the elevated SREBP-1c protein levels (Figures 5C,D). The qRT-PCR results showed that WAC decreased the expression of the lipid synthesis genes *FASN*, *ACC*, *SREBP-1c* and *SCD-1* caused by the high-fat diet (Figure 5E). These results suggest that WAC activates PI3K/AKT and AMPK and decreases SREBP-1c expression.

### 3.6 WAC activates PI3K/AKT and AMPK *in vitro*

**FIGURE 6:**
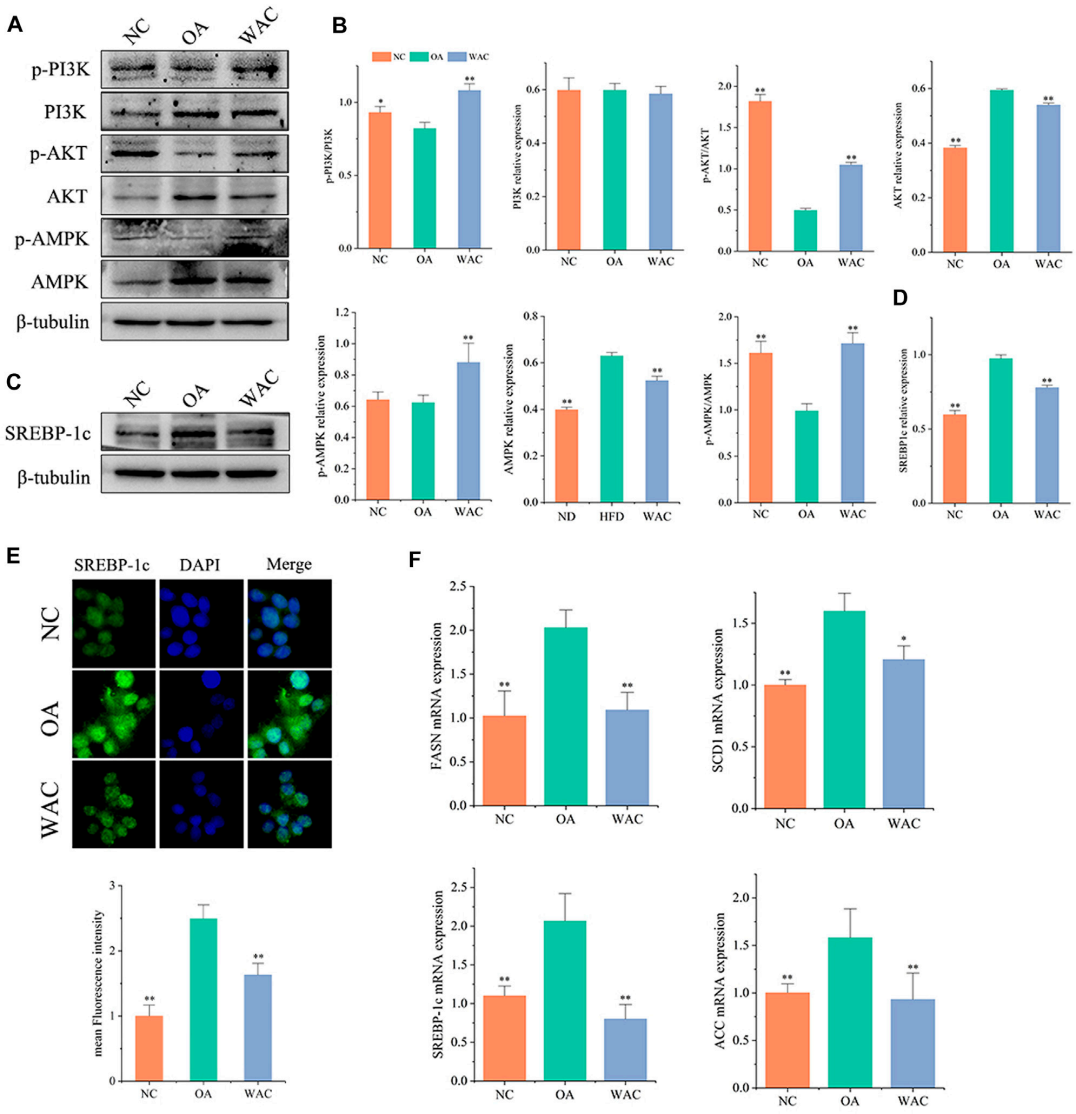
Effect of WAC on OA-induced PI3K, AKT, AMPK phosphorylation and SREBP-1c protein expression in HepG2 cells. **(A,B)** Phosphorylation of PI3K, AKT, AMPK and total protein levels in HepG2 cells. **(C,D)** Protein levels of SREBP-1c in HepG2 cells. **(E)** Graph of SREBP-1c immunofluorescence results. **(F)** mRNA levels of FASN, ACC, SREBP-1c and SCD-1 in HepG2 cells. Compared with OA, * indicates *p* < 0.05 and ** indicates *p* < 0.01.

*In vitro*, total PI3K and AKT and their phosphorylation levels, AMPK and p-AMPK protein levels were measured by protein blotting, and the trends were the same as *in vivo*. The phosphorylated PI3K and AKT protein levels were decreased, p-PI3K/PI3K levels were decreased, AMPK protein levels were significantly increased and p-AMPK protein levels were decreased in HepG2 cells of OA group. After WAC treatment of HepG2 cells, the above results were reversed (Figures 6A–D). As with the results of *in vivo* experiments, SREBP-1c protein levels were significantly elevated in HepG2 cells in the OA group compared with the NC group, and WAC treatment reversed the elevated SREBP-1 protein levels (Figures 6C,D). Immunofluorescence staining of OA and WAC-treated cells using SREBP-1c antibody showed that WAC significantly reduced SREBP-1c expression in HepG2 cells (Figure 6E). WAC decreased OA-induced mRNA expression of the elevated expression of the lipid synthesis genes *FASN*, *ACC*, *SREBP-1c* and *SCD-1* (Figure 6F).

### 3.7 WAC reduces lipogenesis in HepG2 cells

**FIGURE 7:**
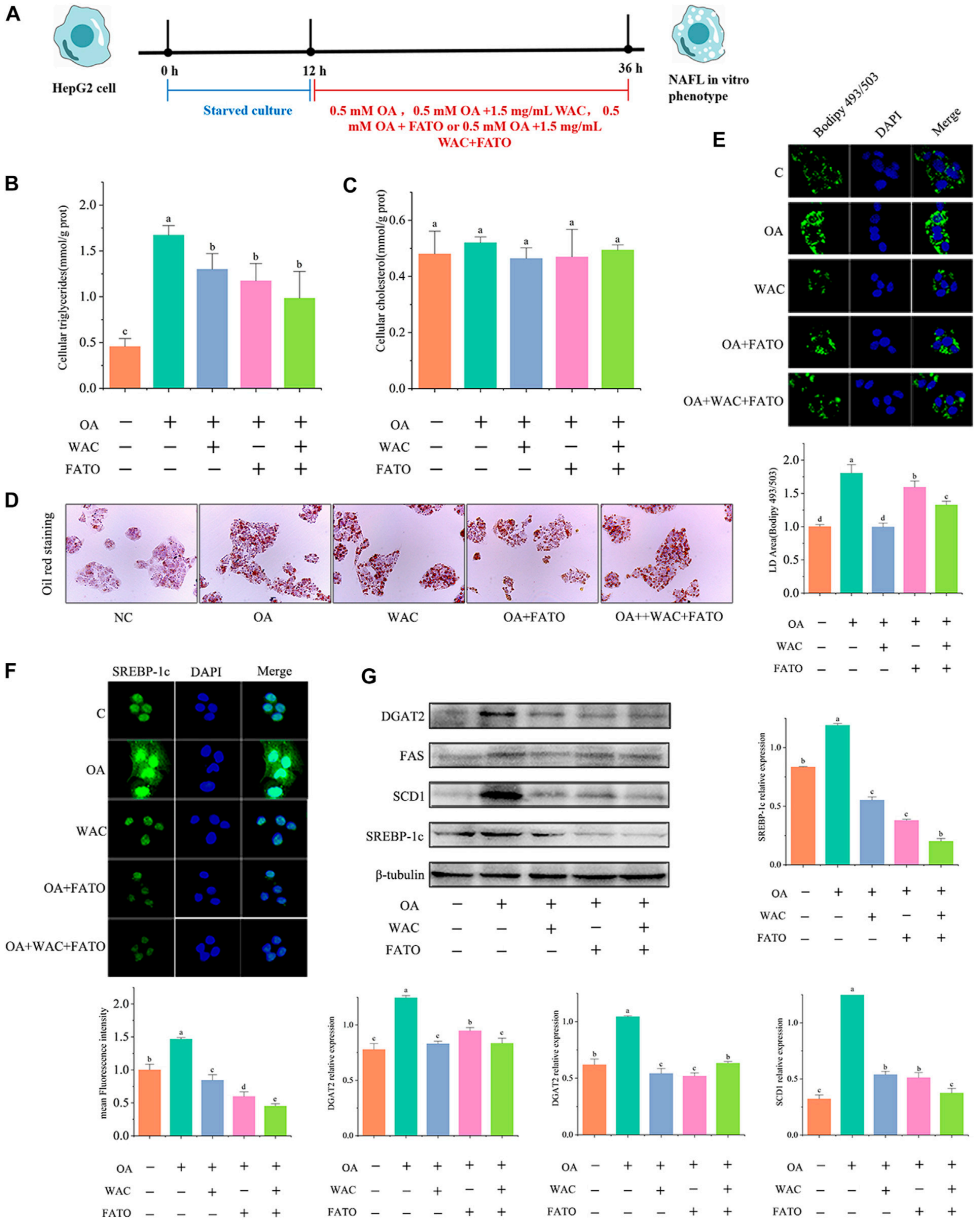
Effect of WAC on SREBP-1c in HepG2 cells. **(A)** Experimental procedure of cells after the addition of FATO. **(B,C)** Triglyceride and cholesterol levels in HepG2 cells. **(D)** Graph of oil red O staining results. **(E)** Graph of BODIPY staining results. **(F)** Graph of SREBP-1c immunofluorescence results. **(G)** SREBP-1c, SCD1, FAS, DGAT2 protein levels in HepG2 cells. Different letters represent significant differences.

We further verified whether SREBP-1c acts as a target of WAC to reduce lipid synthesis. We treated HepG2 cells with the SREBP-1c inhibitor Fatostatin (FATO) *in vitro* and performed subsequent experiments (Figure 7A). After WAC treatment of HepG2 cells, TG was significantly reduced, but TC remained unchanged significantly (Figures 7B,C). Oil red staining and BODIPY493/503 staining also demonstrated that WAC reduced the number of lipid droplets and lipid accumulation (Figures 7D,E). The expression of adipogenesis-related proteins SCD1, FAS and DGAT2 was also examined, and WAC reduced the protein expression levels of SCD1, FAS and DGAT2 compared with the model group (Figure 7G). With the SREBP-1c inhibitor FATO, the trend of action was the same as that of WAC. The above results suggest that WAC reduces lipogenesis in HepG2 cells and is able to regulate the SREBP-1c pathway.

## 4 Discussion

HFD-fed C57BL/6 mice for 16 weeks were successfully constructed as a model of NAFLD. WAC intervention reversed the abnormal levels of TG, TC, GLU, HDL, LDL in serum and TG and TC in liver of mice induced by high-fat diet, and the results of liver pathology tests indicated that WAC reduced the number of hepatic lipid droplets. The results of *in vitro* experiments were also similar, indicating that WAC has the effect of promoting lipid metabolism and reducing the accumulation of lipids in the liver. By using LC/MS technology, Zhao et al. detected nine Nine chlorogenic acid analogues of AC, including chlorogenic acid, cryptochlorogenic acid, neochlorogenic acid, 3,5-dicaffeoylquinic acid, 4,5-dicaffeoylquinic acid, 3,4-dicaffeoylquinic acid, chlorogenic acid methyl ester, cryptochlorogenic acid methyl ester, neochlorogenic acid methyl ester (Zhao et al., 2014). The active components of WAC were identified by LC-MS. Recent studies have found that chlorogenic acid (CGA) inhibits adipocyte differentiation and triglyceride (TG) accumulation (Liu et al., 2021), and caffeic acid (CA) reduces gene expression related to fatty acid synthesis and improves hepatic steatosis (Kim et al., 2018), therefore, we hypothesize that the active substances of WAC that promote lipid metabolism are chlorogenic acid analogues.

PI3K/AKT signaling pathway plays an important role in the development of NAFLD (Huang et al., 2018; Santoleri and Titchenell, 2019). Insulin is recruited through insulin receptor substrate (IRS) and activates PI3K. Activated PI3K signals to its downstream target kinase AKT (Osorio-Fuentealba and Klip, 2015), which inhibits GSK3. GSK3 promotes the degradation of mature SREBP-1c (Cross et al., 1995), which mediates lipid metabolism (Lagathu et al., 2006). SREBP-1c is a major regulator of fatty acid synthesis in the liver (Osborne and Espenshade, 2022). It can control the amount of TG *in vivo* by regulating acetyl coenzyme A carboxylase 1 (ACC1), fatty acid synthase (FASN), stearoyl coenzyme A desaturase (SCD-1) and other enzymes or proteins related to fatty acid synthesis (Wang et al., 2015). In mouse liver and *in vitro* cultured HepG2 cells, we observed that HFD and OA impaired PI3K/AKT signaling pathway and up-regulated SREBP-1c protein expression, whereas WAC increased PI3K/AKT phosphorylation and decreased SREBP-1c expression. WAC decreased TG content and reduced lipid deposition in liver and HepG2 cells. Although there are no reports of WAC affecting NAFLD through the PI3K/AKT signaling pathway, it has been shown that chlorogenic acid prevents HFD-induced hepatic steatosis and may achieve this effect by modulating intestinal flora and increasing GLP-1 secretion (Jiang et al., 2022).

AMPK is a member of the serine/threonine kinase family and has the role of SREBP-1c, a factor that regulates energy homeostasis (Desjardins and Steinberg, 2018). In recent years, the AMPK signaling pathway has been shown to be associated with the alleviation of metabolic disorders in many studies on the treatment of NAFLD. Activation of AMPK signaling pathway is a common feature of NAFLD treatment (Chen et al., 2019; Zhou et al., 2021; Chen et al., 2022; Kim et al., 2022; Liao et al., 2022; Zhang and Feng, 2022). Activated AMPK signaling pathway in the liver reduces NAFLD mainly by reducing lipid production in the liver and regulating fatty acid oxidation in the liver (Day et al., 2017; von Loeffelholz et al., 2021). AMPK is an upstream kinase of SREBP-1c (Zhao et al., 2022). It can inhibit the expression of genes related to lipid synthesis such as SREBP-1c (Kim et al., 2020), and reduce hepatic TG synthesis. In this experiments, p-AMPK/AMPK and SREBP-1c protein expression were detected by western blotting. The results showed that WAC reversed the trend of decreased p-AMPK/AMPK protein expression and increased SREBP-1c expression in HFD mouse hepatocytes and OA-induced HepG2 cells. This indicated that the AMPK/SREBP-1c pathway was activated by WAC. The mRNA expression of the lipid synthesis genes FASN, ACC, SREBP-1c and SCD-1 was also observed by qRT-PCR. This showed that WAC significantly reduced the expression of the above lipid synthesis genes. In summary, WAC reduces TG synthesis in liver and HepG2 cells by activating PI3K/AKT pathway and AMPK to decrease SREBP-1c expression and alleviate hepatic steatosis.

SCD1, FAS and DGAT2 are all downstream factors of SREBP-1c. FAS and SCD1 are known to be involved in the *ab initio* synthesis, esterification and desaturation of fatty acids. DGAT2 catalyzes the final step of TG production and accelerates hepatic lipid synthesis (Liu et al., 2012; Shimano and Sato, 2017). We found that AMPK could regulate the expression of SREBP-1c. To verify whether SREBP-1c is the target of WAC action, we further inhibited SREBP-1c with inhibitors *in vitro* and found that the results after inhibition were consistent with the trend of WAC action results. The expression of SCD1, FAS, and DGAT2 were all inhibited after inhibition of SREBP-1c. The above results indicate that SREBP-1c is the pathway of WAC action, and WAC can regulate the SREBP-1c pathway and reduce lipogenesis in HepG2 cells.

Our experiments showed that WAC can activate PI3K/AKT pathway and AMPK and thus reduce hepatic TG synthesis, but there are some limitations. In this experiment, we did not investigate the specific relationship between PI3K/AKT pathway and AMPK each with SREBP-1c expression by further experiments. This is the focus of our follow-up study.

**FIGURE 8:**
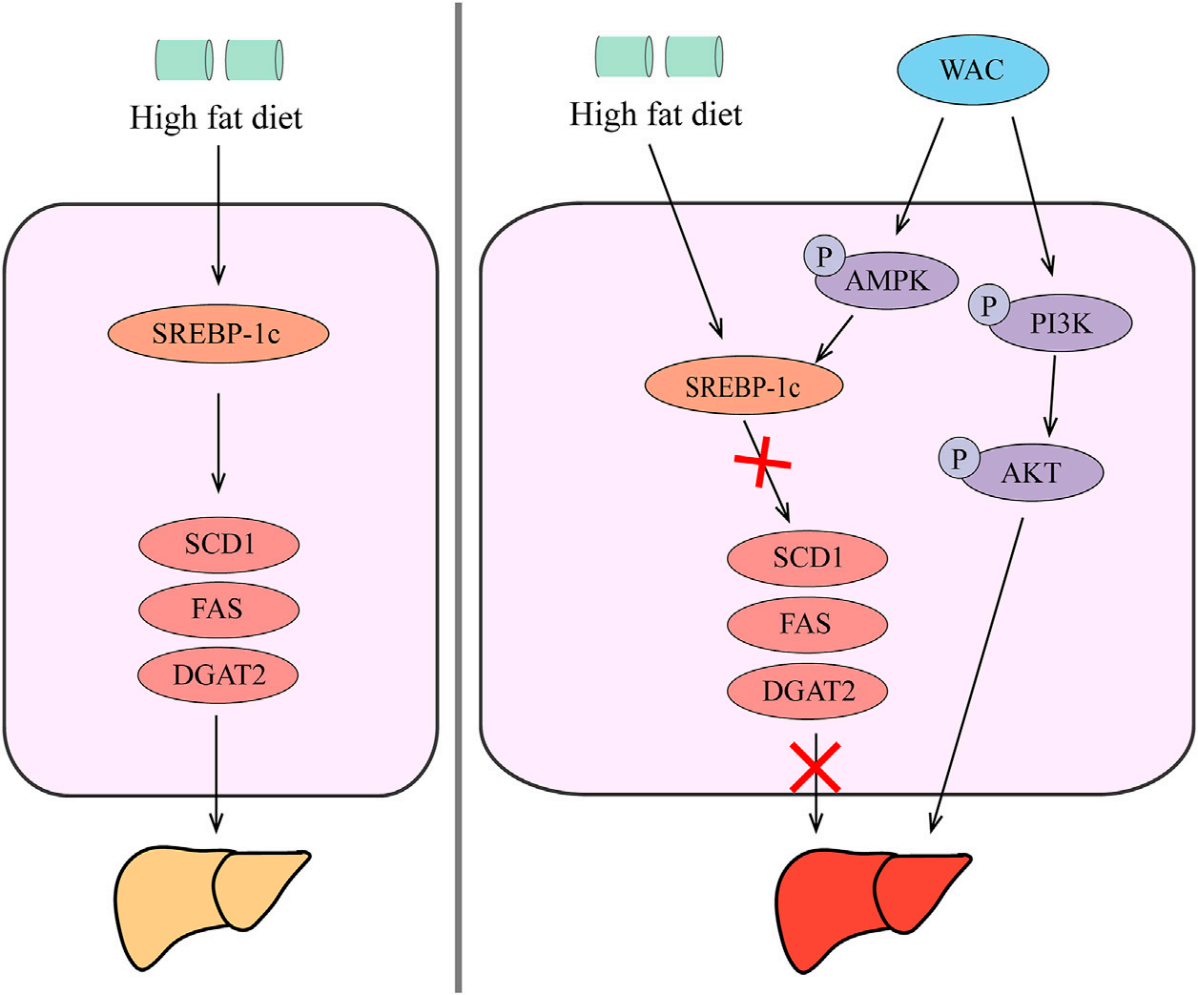
Schematic diagram of the mechanism by which WAC improves NAFLD. WAC activates PI3K/AKT pathway, activates AMPK to promote SREBP-1c expression, thereby reducing hepatic TG synthesis and lipid synthesis.

In summary, our *in vivo* and *in vitro* studies showed that WAC activates PI3K/AKT pathway, activates AMPK to promote SREBP-1c expression, thereby reducing hepatic TG synthesis and lipid synthesis (Figure 8). Therefore, AC could be used as a potential health herb for improving NAFLD and obesity.

## Data Availability

The raw data supporting the conclusions of this article will be made available by the authors, without undue reservation.
