# Supplementary material for: Aqueous extract of Artemisia capillaris improves non-alcoholic fatty liver and obesity in mice induced by high-fat diet
Source: Front Pharmacol. 2022 Nov 28;13:1084435. doi: 10.3389/fphar.2022.1084435 (PMC9742474; doi:10.3389/fphar.2022.1084435)
Supplement: Supplementary file 2 [file Table1.DOCX]

Non-alcoholic fatty liver disease is a clinicopathological syndrome of hepatic steatosis characterized by excessive accumulation of lipids, mainly triglycerides, in hepatocytes. The incidence of nonalcoholic fatty liver disease has shown a high development worldwide, and it is closely related to other metabolic diseases such as obesity, type 2 diabetes, dyslipidemia and hypertension, making nonalcoholic fatty liver disease a global health issue of concern. Artemisia capillaris is the dried above-ground part of Artemisia capillaris Thunb. or Artemisia scoparia Waldst. et Kit.. It is a common botanical used in traditional medicine for the treatment of liver and gallbladder diseases and has the effect of clearing heat and promoting bile. It mainly contains coumarins, flavonoids, organic acids, volatile oils, terpenoids and other chemical components. In recent years, it has been found that, in addition to its pharmacological effects such as choleretic and hepatoprotective, it also has a variety of pharmacological activities such as clearing heat, anti-inflammatory, analgesic, and regulating lipid metabolism. Based on this, we investigated whether Artemisia capillaris could alleviate high-fat diet-induced NAFLD and obesity through some experiments, and found that Artemisia capillaris could reduce body weight and lipogenesis and has potential as a natural health herb for NAFLD and obesity.
